# Smoking in an Inpatient Psychiatric Unit in Ireland with a “Tobacco Free Campus” policy: the prevalence, the associated factors, the social consequences and what can be done to address this

**DOI:** 10.1192/j.eurpsy.2023.1144

**Published:** 2023-07-19

**Authors:** K. Srikumar, A. Adam, F. Sargaison, M. O’Grady

**Affiliations:** 1 Adult Acute Mental Health Unit, Galway University Hospital; 2 School of Medicine, University of Galway, Co. Galway, Ireland

## Abstract

**Introduction:**

Smoking is highly prevalent in patients with mental health disorders and although most literature describes the physical health impact of smoking, there is little which addresses the poverty and social consequences associated with nicotine addiction. In 2022, Ireland’s HSE (Health Service Executive) published clinical guidelines, regarding smoking cessation in healthcare settings, with special attention to mental health settings.

**Objectives:**

The aim of this study was to assess the current prevalence of smoking among inpatients in a psychiatric unit with a “Tobacco Free Campus” policy in place, and the associated patient factors. We also assess the efficacy at which mental health professionals are addressing smoking in this setting.

**Methods:**

We performed a cross-sectional analysis of all patients admitted to the inpatient psychiatric unit on a single date. All inpatients were interviewed using a standardised format to ascertain smoking history and employment status. Case records were examined to record diagnoses and assess the patient’s inpatient care plan, nursing admission proforma and medical admission proforma. Medication charts were examined to ascertain whether Nicotine Replacement Therapy (NRT) was prescribed to those identified as smokers. Using Microsoft Excel, we analysed the smoking behaviours data gathered, and the identification of smokers and their orientation to the Tobacco Free Campus policy, on admission.

**Results:**

Of the 51 inpatients, 78% (n=40) had an Axis 1 diagnosis according to the DSM-4, 72% (n=37) were unemployed and 67% (n=34) were receiving Social Welfare. 57% (n=29) of inpatients were current smokers. 63% (n=25) of smokers had an Axis 1 diagnosis, 51% (n=19) were unemployed and 53% (n=18) were receiving Social Welfare. Since admission, 52% (n=15) of smokers have been smoking more, and 48% (n=14) have been spending more money on tobacco. 7% of smokers (n=2) started smoking on the unit. 50% (n=9) of smokers receiving Social Welfare were smoking more, with the majority in receipt of long-term disability allowance (n=7). Only 10% (n=3) of smokers were prescribed NRT, with only 1 patient taking NRT. 90% (n=26) of smokers did not have smoking addressed in their care plan. 38% (n=11) had a fully completed smoking history in the nursing admission, while only 14% (n=4) had one in the medical admission.

**Conclusions:**

Despite a Tobacco Free Campus policy, smoking continues to be highly prevalent in an inpatient psychiatric setting. Smoking was particularly prevalent in patients with Axis 1 diagnoses, and in the unemployed. A large proportion increased their smoking on admission, and their expenditure on tobacco. More can be done to identify smokers on admission so as to promote quitting, and in turn, reduce the social consequences related.

**Disclosure of Interest:**

None Declared

